# The impact of temperature on canine Chagas disease transmission risk: A modeling study

**DOI:** 10.1371/journal.pntd.0013498

**Published:** 2025-09-03

**Authors:** Edem Fiatsonu, Sina Mokhtar, Martial L. Ndeffo-Mbah

**Affiliations:** 1 Department of Veterinary Integrative Biosciences, College of Veterinary Medicine and Biomedical Sciences, Texas A&M University, College Station, Texas, United States of America; 2 Department of Epidemiology and Biostatistics, School of Public Health, Texas A&M University, College Station, Texas, United States of America; Advanced Centre for Chronic and Rare Diseases, INDIA

## Abstract

**Background:**

Canine Chagas disease is a vector-borne parasitic disease caused by *Trypanosoma cruzi*. *T. cruzi* is transmitted by triatomine bugs (a.k.a. kissing bugs), an ectothermic host species. Understanding how temperature induces changes in vector traits such as fecundity, egg hatching, molting, and activity frequency is essential for developing predictive models for Chagas disease transmission.

**Methods:**

A mechanistic model based on a Ross–MacDonald framework was developed to capture the temperature-dependent dynamics of *T. cruzi* transmission. Using empirical data on the impact of temperature on triatomine bugs’ life traits, temperature-sensitive parameters were estimated using Bayesian inference approach. These parameters were used to compute a thermal suitability metric, *S(T)*, as an indicator of transmission risk.

**Results:**

The model suggests that *S(T)* peaks at 21.8°C (95% CrI: 17.9–22.0°C) and declines to zero below 16.9°C (95% CrI: 15.3–18.2°C) and above 37.7°C (95% CrI: 36.7–38.6°C). Sensitivity analysis shows that triatomine fecundity, egg hatching, and molting rates exert minimal influence on the thermal optimum, while assuming that triatomine activity frequency is constant across temperature keeps *S(T)* constant between 16.9°C and 37.7°C. This indicates that the effect of temperature on the activity frequency of triatomine is a crucial factor affecting the thermal optimum. Spatial analysis of *T. cruzi* transmission risk across Texas indicates that the highest transmission risk is concentrated in South Texas and the Gulf Coast regions. Moreover, there is high seasonal variation in the transmission risk, with South Texas and the Gulf Coast experiencing higher risk during Spring, whereas elsewhere the risk is highest during Summer.

**Conclusion:**

These findings underscore the critical role of temperature in shaping *T. cruzi* transmission dynamics. The study highlights the urgent need for more species-specific empirical research on how temperature affects vector life history traits. Such insights are essential to refine predictive models of Chagas disease and to develop more effective, targeted vector control strategies. These efforts will be crucial in advancing current initiatives aimed at mitigating the veterinary and public health impacts of canine Chagas disease.

## Author summary

Canine Chagas disease, caused by the parasite *Trypanosoma cruzi* and spread by triatomine (“kissing”) bugs, poses an increasing threat in the southern United States, particularly in Texas. Dogs often serve as surveillance sentinels for Chagas disease, which also can be transmitted to humans. Because Triatomine bugs’ body temperature is highly regulated by external environmental conditions, their biological processes and behaviors are closely linked to ambient temperature. In our study, we combined empirical laboratory data and mathematical modeling to explore how temperature influences key traits of triatomine bugs – such as egg production, hatching rate, development, and activity levels – that determine their capacity to transmit the parasite. Our model, based on a classical Ross–MacDonald framework, calculates a temperature-dependent indicator of *T. cruzi* transmission risk. The results indicate that transmission risk peaks at an optimal temperature of around 21.8°C. The potential for *T. cruzi* transmission drops dramatically at temperatures below 17°C and above about 38°C. Spatial analysis across Texas shows that *T. cruzi* transmission is highly seasonal, with regions like South Texas and the Gulf Coast experiencing the highest risk of canine chagas disease outbreaks. This study highlights the crucial role of temperature in driving *T. cruzi* transmission dynamics and underscores the need for temperature-informed vector control strategies. By improving our understanding of how environmental conditions affect disease spread, these findings can help guide targeted interventions and inform public health policies. Future research focusing on species-specific responses to temperature will further refine these models and aid in managing the impact of *T. cruzi* infection on both animal and human health.

## Introduction

Canine chagas disease is a vector-borne disease in dogs, caused by the protozoan parasite *Trypanosoma cruzi* and transmitted by hematophagous triatomine bugs, commonly known as ‘kissing bugs’ [1,2]. Dogs typically become infected by ingesting the feces of infected triatomines. These bugs generally feed on dogs while they are sleeping and often defecate on or near the bite site. Dogs may then ingest the contaminated feces while licking these bite wounds. In addition to fecal-oral transmission, dogs can also acquire the infection by consuming infected triatomines directly [2–6]. *T. cruzi* infection in dogs can lead to cardiac and gastrointestinal complications, although many infected dogs are likely to remain asymptomatic [7,8]. The parasite can also infect humans, with Chagas disease affecting more than 6 million people – primarily in the Americas – where it poses a significant public health concern [9,10].

Human exposure to triatomine bugs is primarily associated with poor housing conditions [9]. In the Southern United States, Chagas disease remains predominantly a canine concern, as improved housing reduces human contacts with the vector. Recent surveillance studies in Texas report canine seroprevalence rates ranging from 7% to over 20% [2,6,7,10], indicating substantial exposure of dog populations to infected triatomines. The distribution of triatomine bugs appears more widespread than previously recognized, prompting critical questions about the environmental factors influencing transmission risk. Although autochthonous *T. cruzi* transmission has been documented in parts of the U.S., dogs continue to serve as the primary domestic reservoir and a key surveillance sentinel for monitoring transmission [4,5,11].

Temperature is one of the most critical environmental factors influencing the life history traits (LHTs) of ectothermic organisms, including triatomine bugs [12,13]. These vectors comprise several species such as *Triatoma infestans*, *Rhodnius prolixus*, and *Triatoma sanguisuga*. Experimental studies demonstrate that triatomines exhibit optimal developmental rates, increased feeding frequency, and enhanced survival within a range of 25°C and 30°C, although thermal optima vary across species [13–18]. Key LHTs – including fecundity, egg hatching, and molting rates – generally increase with rising temperature until declining at extreme thermal thresholds [13,14,19,20]. In addition to shaping vector biology, ambient temperature also influences *T. cruzi* replication within the insect host, with elevated temperatures typically accelerating parasite proliferation [16].

Microclimatic conditions within domestic environments can differ markedly from ambient outdoor temperatures. Structural features such as thatched roofs and insulated walls may buffer temperature extremes, creating stable microhabitats that promote triatomine survival and reproductive success [21]. Field observations in east-central Texas have shown that *T. sanguisuga* dispersal activity is positively correlated with higher ambient temperatures, with peak activity occurring during the warmer months [22,23]. Additionally, flight dispersal in *T. infestans* typically initiates when temperatures exceed 17–18°C [24], directly influencing the risk of canine exposure to infected triatomine bugs.

Mathematical models that incorporate temperature-dependent parameters offer a robust framework for predicting transmission risk. Classical infectious disease models [8,25,26] have recently been enhanced with nonlinear thermal response functions to estimate the temperature-dependent basic reproduction number, *R*_*0*_ *(T)*. In the context of Chagas disease, advanced mechanistic models now integrate detailed laboratory data—including fecundity, egg hatching success, molting efficiency, and mortality rates—with field observations of vector dispersal [27,28]. Additionally, the use of advanced machine learning techniques has refined the experimental identification of thermal thresholds in triatomine vectors, thereby substantially improving the precision and predictive power of these transmission models [29].

As global temperatures continue to rise, understanding how temperature influences *T. cruzi* transmission is critical to anticipating future risks of canine Chagas disease in the southern United States. This study presents a thermally sensitive mechanistic model that integrates key biological and ecological parameters to generate a temperature-dependent indicator of transmission risk. To accomplish this objective, 1) the authors developed a mechanistic model of *T. cruzi* transmission in dog populations based on empirical data, 2) quantified the effects of temperature on a transmission risk indicator, 3) analyzed the relative contribution of each life history trait on this indicator, and 4) assessed the spatial and seasonal dynamics of canine Chagas disease risk in Texas. Our findings provide essential insights to inform targeted disease surveillance, guide vector control strategies, and help mitigate the growing veterinary and public health burden of Chagas disease in the region.

## Methods

### Model description

We developed a mechanistic model for canine chagas disease transmission in settings such as the southern United States, where triatomine species are primarily non-domestic. The model describes the transmission dynamics of *T. cruzi* between triatomine vectors and canine population. Our model is structured as a system of ordinary differential equations (ODEs) with temperature-dependent parameters. The model extends a Ross MacDonald-like model which was previously used for canine chagas disease [28]. The model consists of three equations describing the dynamics of the proportion of infected dogs (*X*), infected triatomine (*Y*), and the ratio of triatomine to dogs (*m*) is given as follows:


$$\frac{dX}{dt}=ma(T)bY(1-X)-rX$$

$$\frac{dY}{dt}=a(T)cX(e^{-gn}-Y)-gY$$

$$\frac{dm}{dt}=R(T)m(1-\frac{m}{K})-gm$$

Where *r* is the dog natural mortality rate, *a* is the bite rate, *b* is the transmission efficiency from infected triatomine to susceptible dog, *c* is the transmission from dog to triatomine, *n* is the extrinsic incubation period, *g* is the triatomine death rate, *b* is the transmission from triatomine to dog, *K* is the relative carrying capacity of triatomine, and *R* is the triatomine relative growth rate. We assume that the triatomine growth rate $R(T)=\theta_{v}(T)\phi_{v}(T)\sigma_{v}(T)/g$ consists of, *θ*_*v*_ the number of eggs produced by a triatomine per day; *ϕ*_*v*_, the probability of egg hatching; and *σ*_*v*_, the probability of surviving from egg to adult, *g*, the triatomine death rate. The contact rate *a* equals $a(T)=a_{0}f(T)$, where $a_{0}$ is the baseline expected number of bites on dogs per triatomine, and f(T) is the temperature preference activity frequency for triatomine.

### Calculation of the basic reproduction number ($R_{0}$) and thermal suitability metric S(T)

To quantify the transmission risk of *T. cruzi,* we employ the Next Generation Matrix (NGM) approach to derive an analytical expression for the basic reproductive number, $R_{0}(T).$

Following the NGM approach, we first partition the system into new *T. cruzi* infection terms (F) and transition (loss) terms (V). For *T. cruzi* infection compartments associated with dogs (X) and Triatomine bugs (Y), we write:

$\frac{dX}{dt}=F_{x}-V_{x}$ and $\frac{dY}{dt}=F_{y}-V_{y}$

Based on our model, we identify:

F = $\left[\;ace^{-gn}0\;\;\;0abm^{*}\right]$, V = [ 0r  g0], where $m^{*}\;$denotes the equilibrium ratio of triatomine bugs to dogs.

Thus, the NGM is given by: $FV^{-1}=\;\left[\;\frac{ace^{-gn}}{r}0\;\;\;0\frac{m*ab}{g}\right]$.

The characteristic equation is obtained by solving $det(\text{F}V^{-1}-\lambda I\;)=\text{0}$, which leads to the spectral radius $R_{0}=\sqrt{\frac{m*a^{2}bce^{-gn}}{rg}}$,

m* = K*(1−g/R); where $R=\theta_{v}\phi_{v}\sigma_{v}/g$


$${\text{R}}_{0}=\sqrt{\frac{K{(1-g^{2}/\theta_{v}\phi_{v}\sigma_{v}\;)a}^{2}bce^{-gn}}{rg}}$$

The temperature-dependent formulation of $R_{0}$ is defined as follows:

$R_{0}(T)=\sqrt{\frac{K{\left(1-g^{2}/(\theta_{v}(T)\phi_{v}(T)\sigma_{v}(T))a(T\right)}^{2}bce^{-gn}}{rg}}$. We can rewrite $R_{0}(T)$ as $R_{0}(T)={\left(\frac{K}{r}\right)}^{1/2}S(T)$ where S(T) is the thermal component of $R_{0}(T)$ and is defined as the suitability metric for the risk of canine disease transmission, with


$$S(T)=\sqrt{\frac{{\left(1-g^{2}/(\theta_{v}(T)\phi_{v}(T)\sigma_{v}(T)\;)){a_{0}}^{2}f(T\right)}^{2}bce^{-gn}}{g}}$$

To investigate the impact of temperature on the risk of canine chagas transmission, we focus on the impact of temperature on the thermal suitability metric. This suitability metric is a density-independent metric that unlike $R_{0}$ is simply an indicator of transmission risk rather than a measure of disease transmission. A similar approach has been used in previous studies [30,31].

To capture spatial heterogeneity in the risk of canine chagas transmission, we used the gridded Climatic Research Unit (CRU) Time-series (TS) data version 4.08: This dataset contains month-by-month climate variations from 1901 to 2023. The data is provided on high-resolution grids with a resolution of 0.5x0.5 degrees. It includes near-surface mean temperature (Celsius) data and was produced by CRU at the University of East Anglia.

### Model parameterization

To accurately capture the temperature-dependent dynamics in our transmission model, we conducted a literature review using the Web of Science. Our search focused on peer-reviewed studies without anytime or geographical constraint. These studies reported empirical data on how temperature impacts key life history traits (LHTs) of triatomine bugs. We found 110 papers matching keywords: (“temperature”, OR “thermal response” OR “thermal sensitivity”) AND chagas disease AND triatomine bugs. We identified only three papers that reported laboratory thermal experimental studies on triatomine fecundity, egg hatching, molting, and triatomine activity, that considered at least four different temperature values [13,16,32]. The first study investigated the effects of temperature on the development of early stages of *Triatoma brasiliensis* and analyzed the thermal preference of nymphs and adults [13]. The second study investigated the effects of different temperatures on *Rhodnius prolixus* development and life cycle, and its relationship with *T*. *cruzi*, concentration in *R. prolixus* urine/feces [16]. The third study investigated the effects of temperature on *Triatoma infestans* and *Triatoma spinolai* activity patterns [32]. For each of these thermally sensitive parameters, we compiled the data from empirical studies (when available) and estimated the best-fitting curve using a Bayesian inference approach. To model the thermal performance curves (TPCs), we have used the bayesTPC package in R [33]. This package provides Bayesian inference for TPCs, allowing for robust parameter estimation and uncertainty quantification. We have used the bTPC() function from the bayesTPC package to fit different thermal performance curve (TPC) models to our data. This function uses Markov Chain Monte Carlo (MCMC) methods via the nimble backend to estimate posterior distributions of model parameters. The models fitted include Gaussian, Briere, quadratic, Ratkowsky. Each model was parameterized according to its theoretical formulation. The Gaussian function is defined as $r_{max}exp(-0.5({\left|T-T_{opt}|/a\right)}^{2})$, the Briere function as $qT(T-T_{min}sqrt\left(T_{max}-T\right)$, the quadratic function as $-q(T-T_{min})(T-T_{max})$, and the Ratkowsky function as $(a(T-T_{min}){\left(1-exp(b(T-T_{max})))\right)}^{2}$. *T* is temperature (°Celsius), $T_{min},T_{max},\;T_{opt}$ represents the minimal, maximal, and optimal temperature, respectively. To determine the most appropriate model for each trait, we calculated the Widely Applicable Information Criterion (WAIC) for each fitted model. WAIC is a fully Bayesian criterion that estimates out of sample predictive accuracy, balancing model fit and complexity. Models with lower WAIC values indicate better predictive performance. For each trait, we selected the model with the lowest WAIC value, as summarized in S1 Text. This approach ensures that the selected models provide a balance between goodness-of-fit and model complexity.

For the constant (temperature-independent) parameter, we used fix point values informed by the literature (Table 1).

**Table 1:** Temperature-independent parameters of suitability metric.

Parameter	Description	Value	Source
*n*	Extrinsic incubation period (day)	45	[34]
*c*	Transmission from dog to triatomine	0.308	[35]
*a*_*0*_	Baseline expected number of bites on hosts per triatomine	1/10	[28]
*b*	Transmission efficiency from infected triatomine to susceptible dog	0.0034	[36,37]
*g*	Triatomine daily death rate	0.001	[28]

## Results

**Fig 1:**
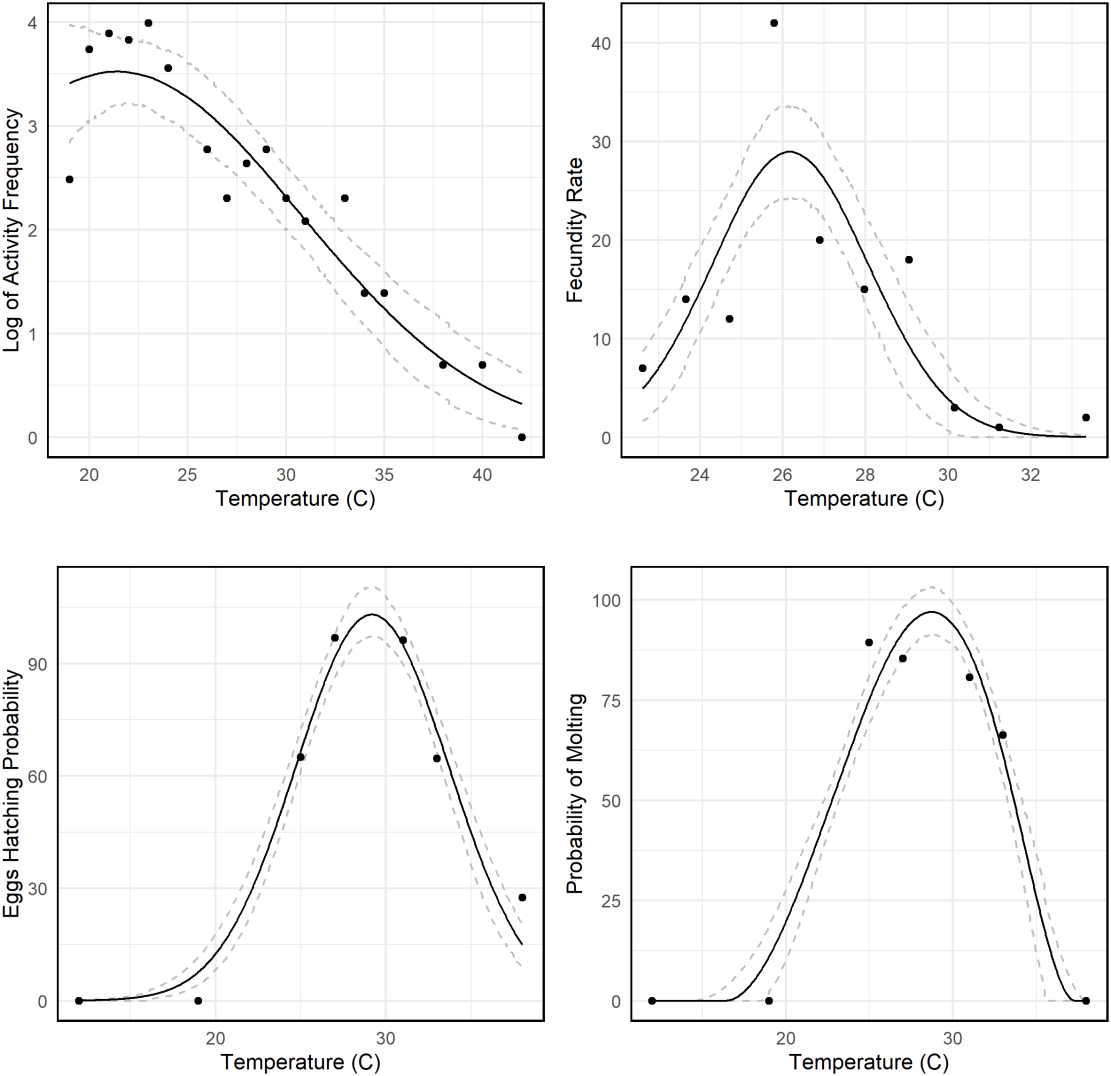
Thermal performance curves for temperature-dependent parameters. The log transform of triatomine activity frequency, log(f(T)), data obtained from [37]; triatomine fecundity rate of 10 female triatomines, 10*$\theta_{v}(T)$, data obtained from [13]; egg hatching probability, $\phi_{v}(T)$, data obtained from [13]; Molting probability, $\sigma_{v}(T)$, data obtained from [13]. Other model variables that were sourced from other studies are referenced in Table 1.

We illustrated the temperature dependence of key triatomine life history traits, each displaying a characteristic unimodal response (Fig 1). The panel in the upper left shows that triatomine activity is minimal at approximately 16°C, increases sharply into the middle to upper 20s, and then declines beyond roughly 28–30°C, suggesting that moderate temperatures optimize host seeking and dispersal while excessive heat imposes stress. Similarly, the panel in the upper right reveals that fecundity, quantified as the number of eggs produced per female per day, is nearly negligible below 20°C, rises steeply to a peak around 26°C, and subsequently diminishes as temperatures exceed 28°C, indicating an optimal thermal window for reproduction. The panel in the bottom left suggests that the probability of successful molting in nymphs is minimal at around 15°C, peaks near 28°C with success rates exceeding 90%, and drops off at temperatures above 30°C, underscoring the critical role of moderate warmth for efficient developmental progression. Collectively, these findings emphasize that triatomine performance is finely tuned to a moderate temperature range, with key biological processes deteriorating under both cooler and excessively warm conditions. In the panel in the bottom right, egg hatching probability is low between 15°C and 20°C, reaches nearly optimal rates (approaching 100%) between approximately 24°C and 28°C, and declines above 30°C, highlighting a narrow “sweet spot” for embryonic development.

**Fig 2:**
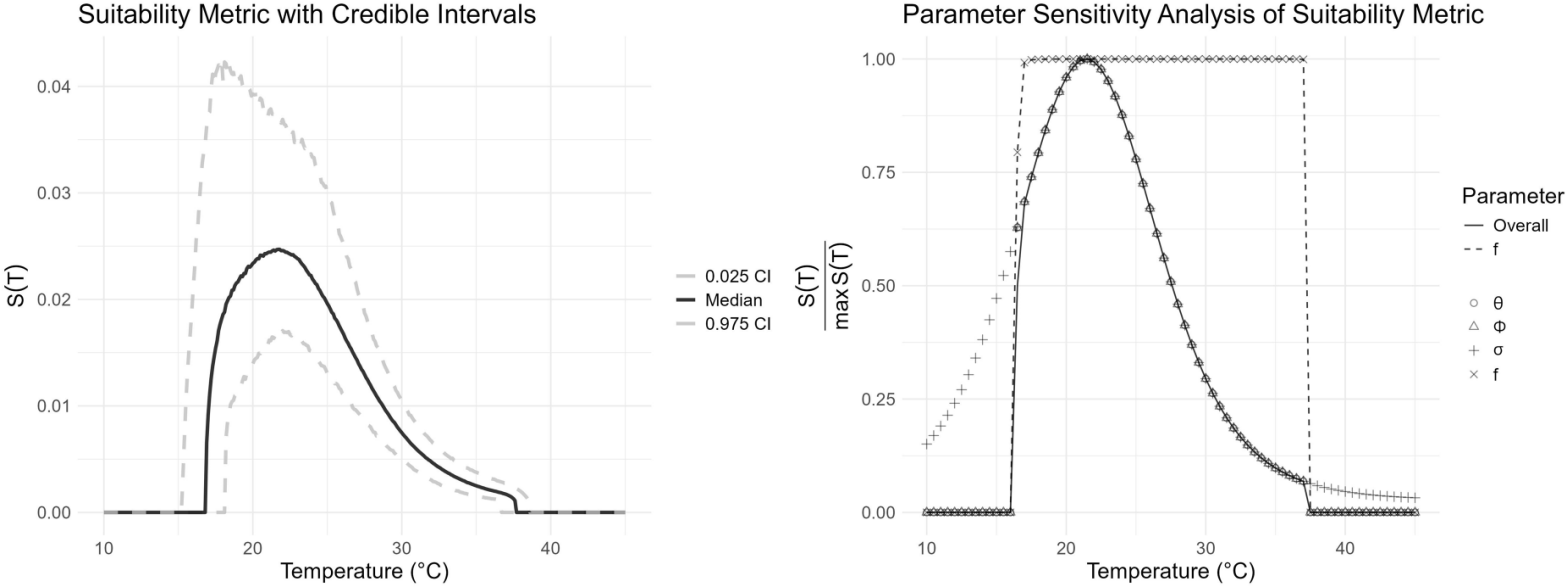
The plot (A) shows the temperature dependent suitability metric, *S(T)*, curve (dark line) with the 95% credible interval (dashed lines), and (B) shows the behavior of *S(T)* assuming the temperature-dependent specific parameter is constant.

We used the posterior distribution of these parameters and our suitability metric formula to estimate the posterior distribution of *S(T)* and infer key temperature values that indicate suitability of *T. cruzi* transmission (Figs 1 and 2A). At constant temperatures, transmission peaks at 21.8°C (95% credible interval (CrI): 17.9 − 22.0°C) and drops to zero at temperatures below 16.9°C (95% CrI: 15.3 – 18.2°C) and above 37.7°C (95% CrI: 36.7 – 38.6°C). We performed a sensitivity analysis on *S(T)* with respect to temperature-dependent parameters (Fig 2B). Specifically, we evaluated the behavior of *S(T)* assuming each parameter to be constant. *S(T)* value appears to remain unchanged when triatomine fecundity rate, $\theta_{v}(T)$, egg hatching probability, $\phi_{v}(T)$, and molting probability, $\sigma_{v}(T)$, are assumed to be constant (Fig 2B). If triatomine activity frequency is constant, then *S(T)* remains constant at its maximum value for temperatures between 16.9°C and 37.7°C, and drops to zero outside of that temperature range (Fig 2B).

### Case of Texas

**Fig 3:**
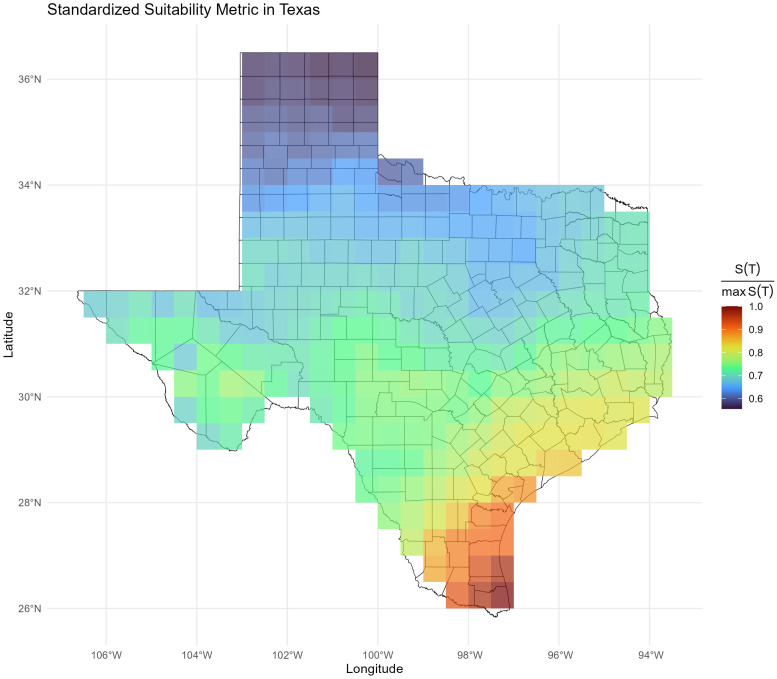
Average annual relative suitability metric values of *T. cruzi* transmission risk in Texas. The average values were computed using temperature data from 2000 to 2023. Shapefile can be obtained from the US Census Bureau at https://www.census.gov/cgi-bin/geo/shapefiles/index.php?year=2024&layergroup=Counties+%28and+equivalent%29.

To investigate the impact of temperature on the risk of canine chagas transmission in areas such as the Southern USA, we focused on the state of Texas where cases of *T. cruzi* infections among dogs have been widely reported [2,6,7]. We computed *S(T)* values at a 5 km x 5 km grid using monthly temperature data from 2000 to 2023 for each grid. Using these monthly *S(T)* values we estimated the average annual *S(T)* relative to its maximum value (Fig 3). For each grid cell and year, we calculated the annual average *S(T)* by taking the mean of the 12 monthly values. We then normalized these annual averages by dividing them by the maximum observed *S(T)* across the state. This produced a relative measure of annual suitability ranging from 0 (least suitable) to 1 (most suitable). This relative *S(T)* value varies between 0 and 1, where 1 indicates the highest annual risk and 0 indicates no annual risk. Our model shows that the average annual risk, *S(T),* is highest in the South Texas and Gulf Coast regions, followed by the Central, East, and South-West Texas regions (Fig 3). The lowest average annual risk is shown to occur in the North and West Texas regions (Fig 3).

**Fig 4:**
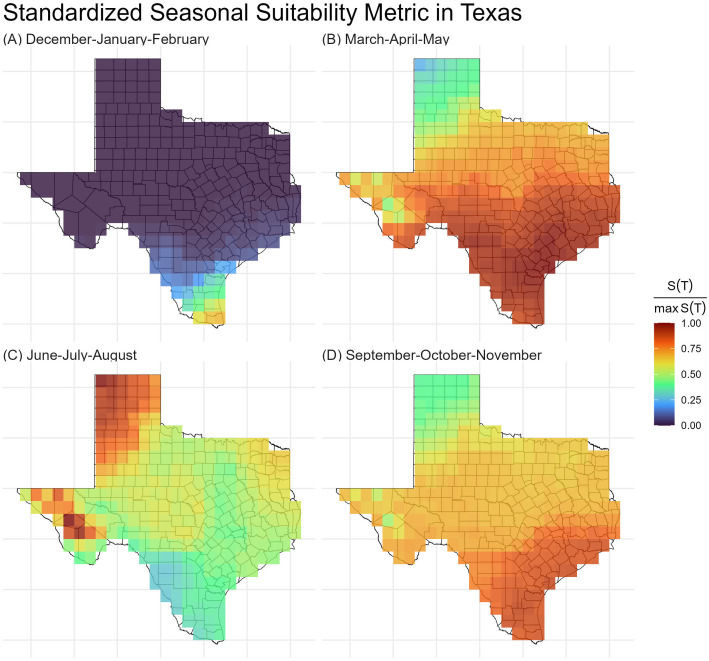
Seasonal average relative suitability metric values from 2000 to 2023, with the time periods corresponding to (A) Winter (December-January-February), (B) Spring (March-April-May), (C) Summer (June-July-August), and (D) Autumn (September-October-November). Shapefile can be obtained from the US Census Bureau at https://www.census.gov/cgi-bin/geo/shapefiles/index.php?year=2024&layergroup=Counties+%28and+equivalent%29.

To investigate the effect of seasonal changes on the risk of canine chagas disease transmission in Texas, we evaluated the mean suitability metric values over the four seasons (Fig 4): Winter (December-January-February), Spring (March-April-May), Summer (June-July-August), and Autumn (September-October-November). Our model shows that the suitability metric is lower across Texas during the Winter months (December-February) (Fig 4A). During Spring, the transmission risk is shown to be highest in the South Texas and Gulf Coast regions (Fig 4B). Though the transmission risk was shown to be high across the state, during Summer, the West and East regions were shown to have the highest risk (Fig 4C). During Autumn, the Gulf Coast and Southern Texas-Mexico border regions were shown to have the highest transmission risk (Fig 4D).

## Discussion

Our study suggests that ambient temperature is a critical driver of *T. cruzi* transmission risk among canine populations. By integrating experimental data on triatomine life history traits and behavior into a mechanistic model, we quantified a thermal suitability metric, *S(T)*, which exhibits a pronounced unimodal response with an optimum near 21.8°C (95% CrI: 17.9–22.0°C) and declines sharply outside the range of approximately 16.9°C to 37.7°C (Figs 1 and 2). Sensitivity analyses indicate that triatomine activity frequency can substantially affect the thermal optimum of *T. cruzi* transmission risk, underscoring the dynamic interplay between vector behavior and the risk of *T. cruzi* infection in dog populations.

Experimental studies form the cornerstone of our research. For example, Guarneri et al. [13] found that *T. brasiliensis* exhibits significantly increased fecundity and egg hatching rates near 26°C, with a marked decline outside this thermal optimum. Similarly, Schilman and Lazzari [14] reported that *R. prolixus* prefers moderate temperatures, aligning with our findings of reduced vector activity and transmission potential at temperatures below approximately 16.9°C and above 37.7°C. Temperature-dependent infectivity is another critical factor. Elliot et al. [15] demonstrated that *T. cruzi* virulence in *R. prolixus* varies with temperature, with replication accelerating at higher but sub-extreme temperatures. Complementing this, Loshouarn and Guarneri [16] showed that parasite load peaks around 28°C. Furthermore, laboratory data on developmental traits—including egg hatching and molting, which are optimized between 24°C and 28°C [13]—along with log-transformed activity frequency data [37], underscore the sensitivity of these biological processes to thermal variation.

Fimbres-Macías et al. [22] documented increased dispersal activity in *T. sanguisuga* in east-central Texas at elevated ambient temperatures, supporting our model’s prediction of heightened transmission risk during the warmer months (June–August). Additional studies [23,24] report that flight dispersal in triatomines typically begins when temperatures exceed 17–18°C, corroborating the thermal thresholds incorporated into our model. Our spatial analysis across Texas, using monthly *S(T)* values calculated over a 23-year period (2000–2023) at a 5 km × 5 km resolution, suggests that the highest annual transmission risk is concentrated in South Texas and along the Gulf Coast, with comparatively lower risk in northern and western regions (Fig 3). Seasonal patterns show the lowest risk during winter (December–February), peaking in spring in South Texas and the Gulf Coast, shifting to elevated risk in western and eastern Texas during summer, and rising again in autumn along the Gulf Coast and the Texas–Mexico border (Fig 4). These spatial and seasonal trends underscore the need for targeted, region-specific vector control strategies to effectively mitigate the transmission of *T. cruzi*.

Similar unimodal thermal responses have been observed in other vector-borne disease systems, including those involving *T. infestans* [20] and arbovirus transmission by *Aedes* mosquitoes [38]. These consistent patterns across diverse systems suggest that our temperature-dependent modeling approach is suitable for this system. The convergence of these findings across various vector-pathogen interactions suggests that the mechanisms captured in our model may be applicable to a wide range of diseases transmitted by ectothermic vectors.

Our findings hold important implications for public health, particularly in regions where dogs serve as early sentinels of Chagas disease transmission. By pinpointing temperature thresholds that optimize parasite spread, this study offers actionable insights for designing targeted surveillance and intervention strategies. In areas with high canine seroprevalence, integrating temperature monitoring into public health practices may enable earlier detection and rapid deployment of vector control efforts, potentially curbing spillover risk to human populations. This fusion of empirical observations with mechanistic modeling deepens our understanding of temperature’s role in disease ecology and supports a proactive, data-informed approach to managing the threat of *T. cruzi* transmission.

### Limitation of the study

A major strength of this study lies in the integration of robust empirical data with advanced Bayesian inference techniques to generate precise thermal performance curves. Nonetheless, several limitations merit consideration. For instance, we assumed the extrinsic incubation period and triatomine mortality rate to be temperature-independent due to a lack of empirical data on their temperature sensitivity, despite existing knowledge indicating that both traits are indeed temperature-responsive. Relaxing this assumption may quantitatively influence our results. To improve model accuracy, additional empirical research is needed to elucidate how temperature affects these key entomological parameters. Moreover, the values for the extrinsic incubation period and transmission rates were derived from guinea pig experiments. Data from dog-based studies are essential to refine parameter estimates for canine-specific transmission dynamics. Our model also did not explicitly include triatomine developmental duration, a factor that could substantially influence *T. cruzi* transmission. This exclusion was due to insufficient data to parameterize a thermal performance curve for development rates across the various nymphal instar stages. Capturing these dynamics will require a more complex transmission model that accounts for egg development, nymphal progression, and the potential contribution of each stage to transmission.

Additionally, the entomological data used in this study were drawn from multiple triatomine species. As shown in arbovirus transmission studies comparing *Aedes aegypti* and *Aedes albopictus* [38], species-specific responses to temperature can differ markedly. While this assumption may impact the quantitative outputs of our model, we expect only minor effects on the qualitative nature of our findings. Nevertheless, species-specific data will be critical for future model refinement, particularly to assess the effects of temperature on species coexistence and differential transmission risk. Beyond entomological and epidemiological considerations, socioeconomic and behavioral factors, as well as proximity and interaction with wildlife, also shape vector exposure and *T. cruzi* transmission. These dimensions were not included in our model to maintain a focused investigation on temperature’s role. Future modeling efforts should aim to incorporate these multifactorial influences into an integrated framework for predicting and managing the risk of canine Chagas disease in the southern United States and beyond.

Due to limited data on how temperature affects the life history traits of triatomine bugs, we employed a suitability metric as a proxy for transmission risk rather than the conventional basic reproduction number, $R_{0}$. The $R_{0}$ metric—defined as the average number of secondary cases produced by a single infectious host in a fully susceptible population—is directly informed by empirical measures such as vectorial capacity and disease seroprevalence [39,40]. To estimate the temperature-dependent reproduction number, R(T), of canine Chagas disease, future research should prioritize the collection of detailed, species-specific thermal performance data for key triatomine traits. These include developmental rates, fecundity, egg hatching success, behavioral activity, incubation periods, mortality rates, feeding frequency, and vector density. Targeted laboratory and field experiments will be essential to validate and extend the findings of this study. Ultimately, such efforts will enhance the accuracy of predictive models and support the development of more effective, temperature-informed vector control strategies—particularly in the context of a rapidly changing environment.

## Supporting information

S1 Text Supplementary methods and results. (S1_Text.DOCX)
