# Supplementary material for: The impact of temperature on canine Chagas disease transmission risk: A modeling study
Source: PLoS Negl Trop Dis. 2025 Sep 3;19(9):e0013498. doi: 10.1371/journal.pntd.0013498 (PMC12419651; doi:10.1371/journal.pntd.0013498)
Supplement: S1 Text — (S1_Text.DOCX) [file pntd.0013498.s001.docx]

**S1_Text: SUPPLEMENTARY METHODS AND RESULTS**

1. **Model Selection and Fitting**

To model the thermal performance curves (TPCs), we have used the bayesTPC package in R. This package provides Bayesian inference for TPCs, allowing for robust parameter estimation and uncertainty quantification.

- 1. **Model Selection**

To determine the most appropriate model for each dataset, we calculated the Widely Applicable Information Criterion (WAIC) for each fitted model. WAIC is a fully Bayesian criterion that estimates out of sample predictive accuracy, balancing model fit and complexity. Models with lower WAIC values indicate better predictive performance. For each dataset, we selected the model with the lowest WAIC value, as summarized in **Table 1**. This approach ensures that the selected models provide a balance between goodness-of-fit and model complexity.

| Dataset | Model | WAIC |
| --- | --- | --- |
| Activity Frequency | briere | 49148.81 |
| **Activity Frequency** | **gaussian** | **27.48** |
| Activity Frequency | quadratic | 45.45 |
| Activity Frequency | ratkowsky | 1513.88 |
| Fecundity Rate | briere | 11007.47 |
| **Fecundity Rate** | **gaussian** | **226.06** |
| Fecundity Rate | quadratic | 343.83 |
| Fecundity Rate | ratkowsky | 805.28 |
| Eggs Hatching Probability | briere | 45411689787.67 |
| **Eggs Hatching Probability** | **gaussian** | **23535.22** |
| Eggs Hatching Probability | quadratic | 19794683.98 |
| Eggs Hatching Probability | ratkowsky | 13941075716700.23 |
| Probability of Molting | briere | 33369.43 |
| Probability of Molting | gaussian | 14860.97 |
| Probability of Molting | quadratic | 25533.99 |
| **Probability of Molting** | **ratkowsky** | **1614.10** |

Table 1: WAIC Scores for Different Models

- 1. Model Fitting

We have used the bTPC() function from the bayesTPC package to fit different thermal performance curve (TPC) models to our data. This function uses Markov Chain Monte Carlo (MCMC) methods via the nimble backend to estimate posterior distributions of model parameters. The models fitted include Gaussian, Briere, quadratic, Ratkowsky. Each model was parameterized according to its theoretical formulation.

- 1. Parameter Estimation

For each fitted model, we extracted the posterior distributions of the model pa- rameters and summarized them using the median and credible intervals (2.5%, 25%, 50%, 75%, and 97.5% quantiles). These posterior summaries provide in- sight into the uncertainty associated with each parameter estimate. The full posterior distributions for each parameter has been attached below.

|  | 2.5% | 25% | 50% | 75% | 97.5% |
| --- | --- | --- | --- | --- | --- |
| *Topt* | 8.12 | 19.57 | 21.43 | 22.53 | 23.91 |
| *a* | 6.81 | 8.34 | 9.39 | 10.93 | 17.37 |
| *rmax* | 3.20 | 3.45 | 3.59 | 3.76 | 5.06 |

Table 2: Posterior Quantiles of Gaussian Model Parameters for Activity Frequency

|  | 2.5% | 25% | 50% | 75% | 97.5% |
| --- | --- | --- | --- | --- | --- |
| *Topt* | 25.78 | 26.05 | 26.17 | 26.31 | 26.61 |
| *a* | 1.40 | 1.72 | 1.90 | 2.09 | 2.49 |
| *rmax* | 23.69 | 27.19 | 29.14 | 31.21 | 35.07 |

Table 3: Posterior Quantiles of Gaussian Model Parameters for Fecundity Rate

|  | 2.5% | 25% | 50% | 75% | 97.5% |
| --- | --- | --- | --- | --- | --- |
| *Topt* | 28.91 | 29.09 | 29.20 | 29.30 | 35.18 |
| *a* | 0.95 | 4.30 | 4.47 | 4.62 | 4.93 |
| *rmax* | 97.47 | 101.42 | 103.66 | 106.12 | 694.04 |

Table 4: Posterior Quantiles of Gaussian Model Parameters for Eggs Hatching Probability

|  | 2.5% | 25% | 50% | 75% | 97.5% |
| --- | --- | --- | --- | --- | --- |
| *Tmax* | 35.18 | 36.88 | 37.34 | 37.83 | 38.44 |
| *Tmin* | 2.83 | 15.44 | 16.34 | 16.90 | 17.92 |
| *a* | 0.35 | 1.24 | 1.62 | 2.44 | 3.47 |
| *b* | 0.03 | 0.05 | 0.08 | 0.11 | 0.63 |

Table 5: Posterior Quantiles of Ratkowsky Model Parameters for Probability of Molting


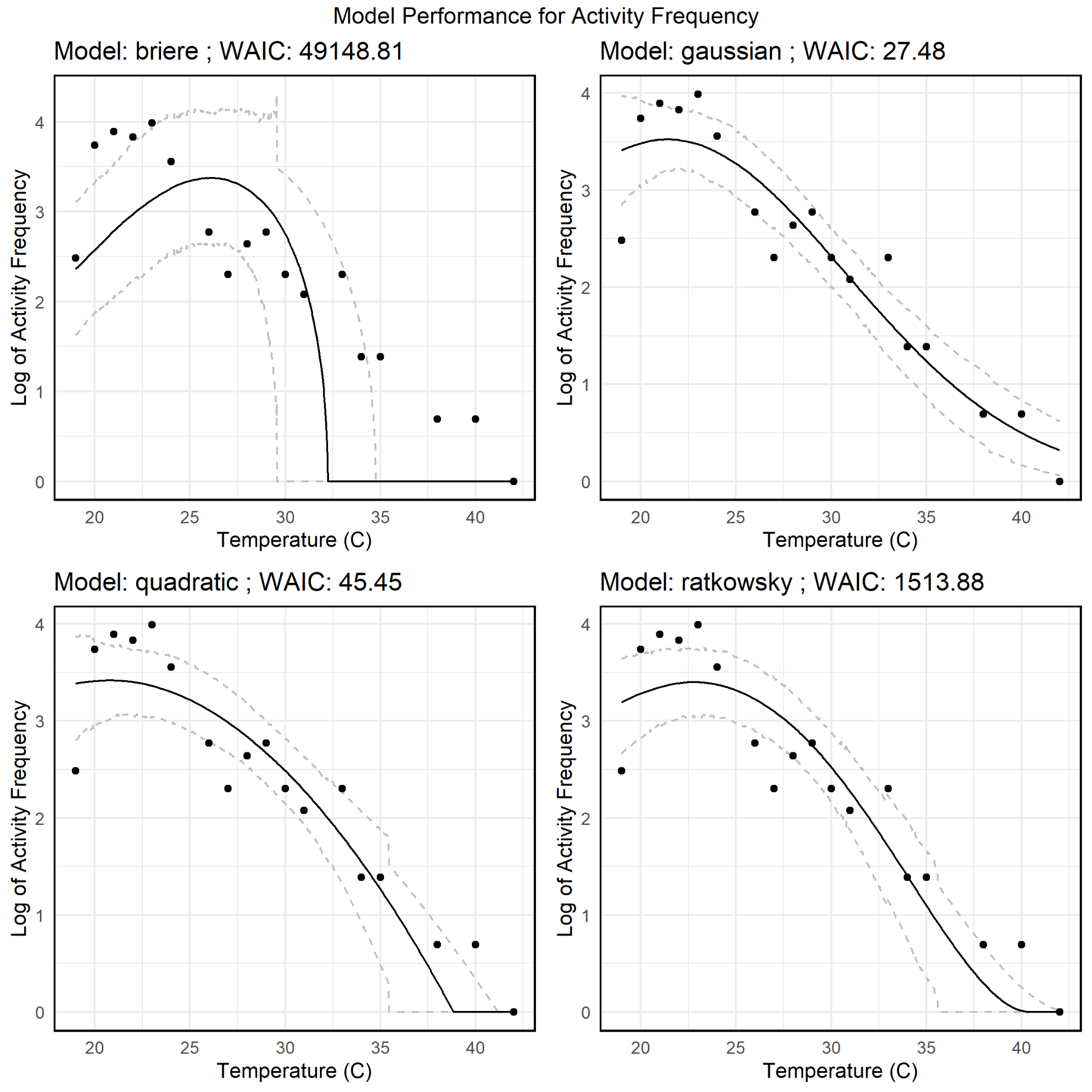


Figure 1: Model performance for activity frequency


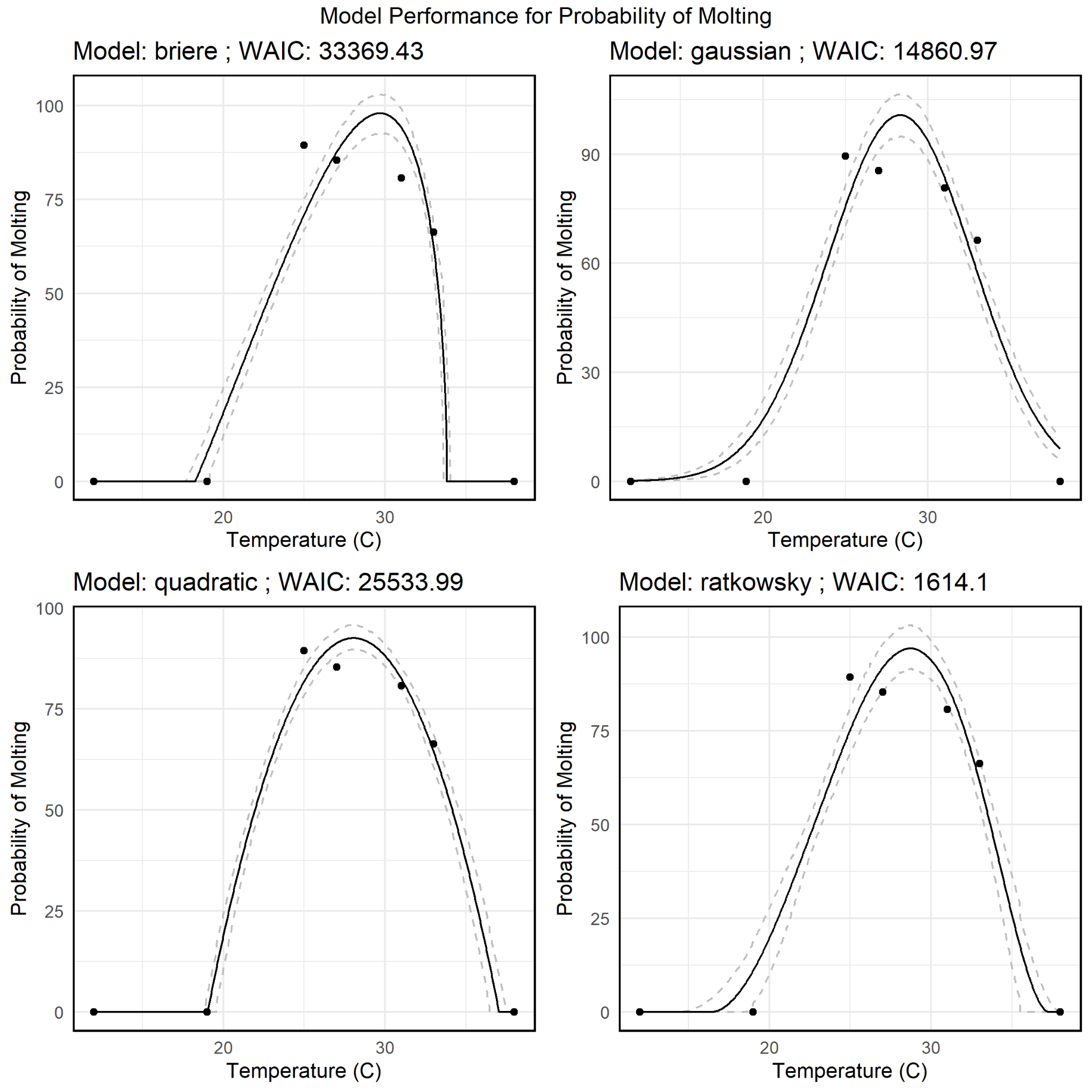


Figure 2: Model performance for probability of molting


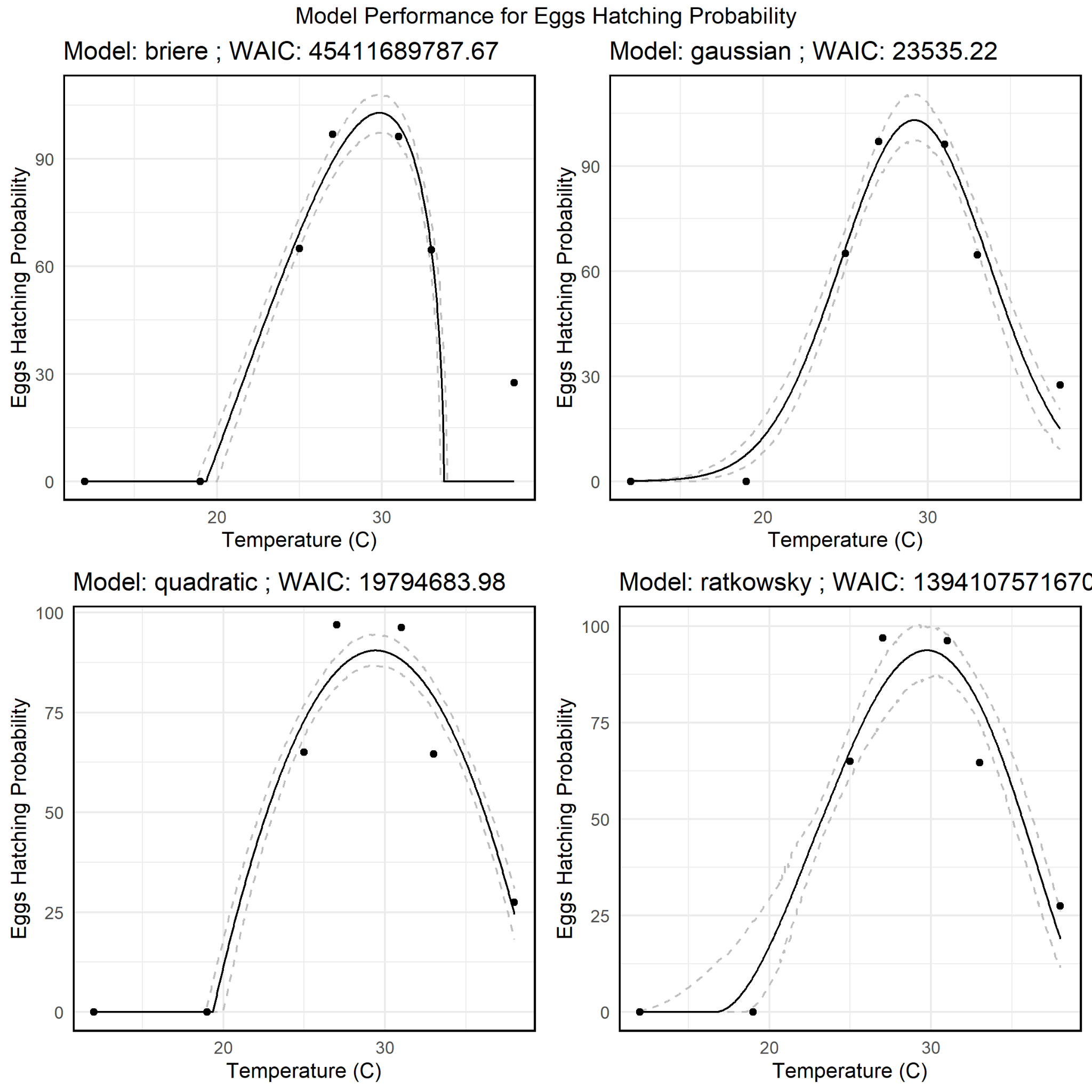


Figure 3: Model performance for egg hatching probability


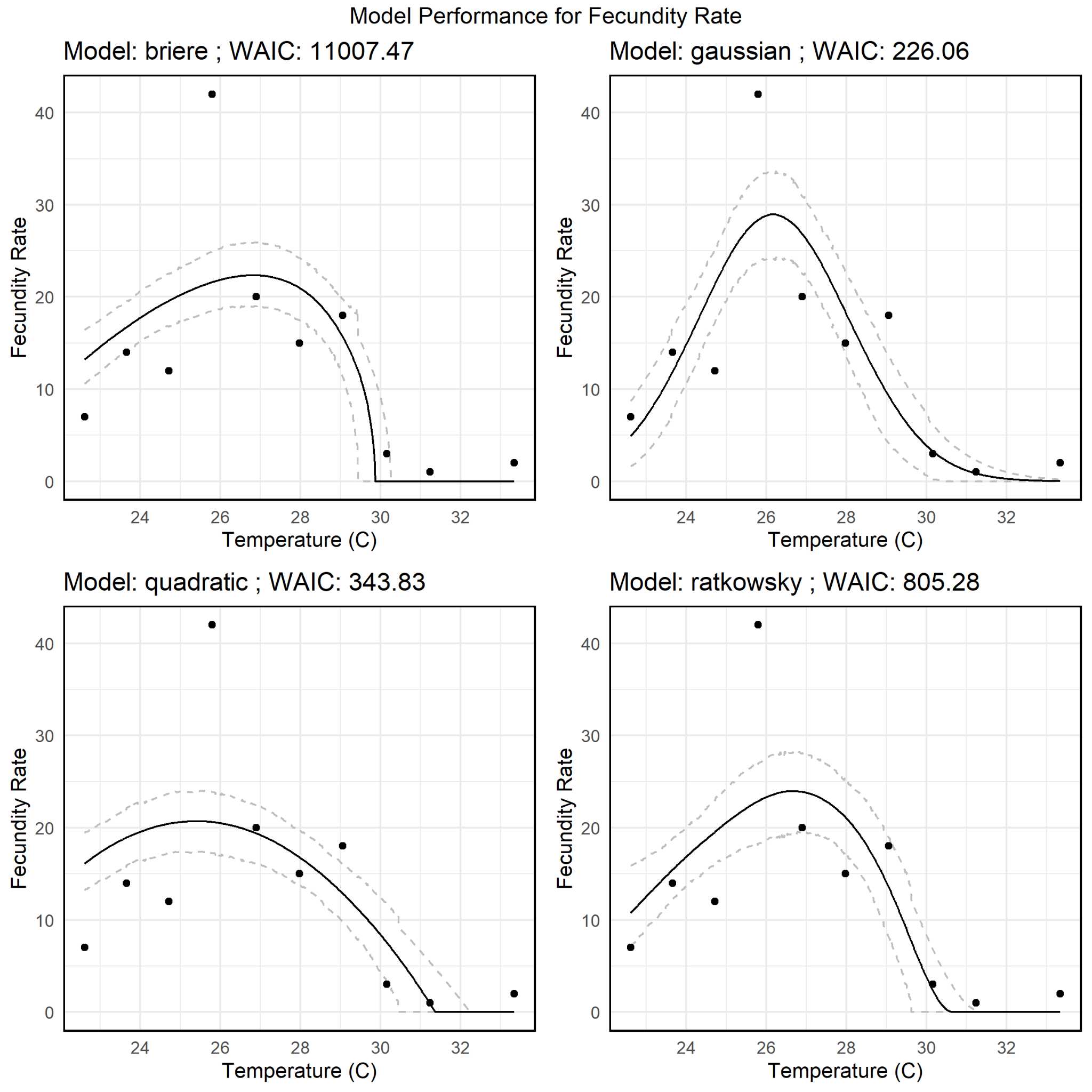


Figure 4: Model performance for fecundity rate
